# Exploring Healthcare Provider Recruitment in a Rural and Frontier Community in Northern Idaho

**DOI:** 10.3390/healthcare12111052

**Published:** 2024-05-21

**Authors:** Jonathan D. Moore, Madeline P. Casanova, Allie M. Lords, Ann V. Lima, Cody Wilkinson, Russell T. Baker

**Affiliations:** 1 Idaho Office of Rural and Underserved Medical Research, University of Idaho, Moscow, ID 83844, USA; jonathandm@uidaho.edu (J.D.M.); russellb@uidaho.edu (R.T.B.); 2 WWAMI Medical Education Program, University of Idaho, Moscow, ID 83844, USA; alords@uw.edu; 3 Department of Family Medicine, University of Washington, Seattle, WA 98195, USA; ann.lima@kh.org; 4 St. Mary’s Health & Clearwater Valley Health, Orofino, ID 83544, USA

**Keywords:** recruitment, NCAQ, frontier, rural, healthcare provider

## Abstract

Limited U.S. research has been conducted examining factors affecting healthcare provider recruitment in rural settings, necessitating community-level investigations due to community differences. The aim of this study was to explore the factors involved in healthcare provider recruitment in a rural community in Northern Idaho. A retooled version of the Nursing Community Apgar Questionnaire (NCAQ) was used to collect data from 50 healthcare providers to assess items influencing provider recruitment. Items were categorized into five factors: geographic, economic, scope of practice, medical support, and facility and community support classes. Healthcare providers ranked items based on perceived importance and how advantageous or challenging it was to recruitment. A “Community Apgar” score is a composite score calculated using the advantage/challenge and importance scores. In our sample, medical support was rated as the most important class. Additionally, facility and community support was rated as the highest advantage class and had the most impactful Apgar scores, meaning it contained the most important advantage and challenge. Our findings suggest that these classes contain dominant factors related to the recruitment of providers in rural areas. Rural healthcare organizations seeking to improve the recruitment of healthcare providers should consider the potential impact of these factors on their population. Further investigations should be conducted on diverse rural samples across the U.S. to enable comparisons of research findings.

## 1. Introduction

Idaho, a predominately rural state [1] with a population of approximately 1.9 million people [2], has severe challenges and limitations related to healthcare professionals. For example, in 2020, Idaho had the fewest physicians per capita of any state, with 196 active physicians per 100,000 people, in comparison to 287 per 100,000 people in the U.S. overall [3]. Idaho also has one of the lowest rates of nurses, with only 7.83 (compared to the overall U.S. rate of 9.19) nurses for every 1000 residents [4]. Additionally, most of the state has health profession shortage areas (HPSAs), with 98.7% of the state designated as HPSAs for primary care, 100% for mental healthcare, and 95.7% for dental healthcare [5].

In addition to the limited number of healthcare workers in Idaho, there are also challenges related to treating individuals living in rural areas. For example, national data have highlighted the challenges healthcare providers in rural areas experience, in particular, the number of roles and duties expected to be completed because they are the sole provider in a remote and/or low-populated area [6]. Researchers have found that the increased burden on physicians in rural areas to hold multiple professional roles in the community and assume care for multiple aspects of a patient’s health can negatively impact their emotional well-being [6,7]. Additionally, in rural areas, anonymity has been cited as a concern for patients seeking and receiving care due to the stigma associated with health conditions/procedures and lack of privacy [8]. Researchers have also suggested that patients in rural areas express more concerns over confidentiality with their providers than patients in urban areas do [9].

Additional constraints exist for healthcare providers working in rural areas, and there are multiple reasons that life as a rural healthcare provider may be less favorable compared to providers working in an urban setting. For example, physicians in rural areas often experience longer workdays and spend more time on call compared to physicians in urban settings [10]. Rural providers also do not have access to as many social events, cultural experiences, or events the lack thereof could negatively impact their personal well-being [10]. Additionally, there is also a lower level of insurance reimbursement in rural medical practice than its urban counterpart, with 70% of payments being from Medicaid, Medicare, or self-pay [10,11]. Other factors have also been identified as affecting provider recruitment in rural regions, including the nursing workforce [12], emergency care [13], telemedicine [14], income [15], financial incentives [16], and spousal support [13].

Strategies have been created and implemented to facilitate and improve the recruitment of healthcare providers. One framework includes three strategic components: (1) information sharing, (2) community engagement, and (3) supporting families/spouses [17]. Information sharing allows prospective providers to learn about the rural area, for example, regarding potential employment opportunities for a spouse or education for a child [17]. Community engagement allows members of the public to help define the strategies of recruitment that are suitable for their community [17]. Lastly, facilitating spousal and family integration in the community with services/opportunities is vital to provider recruitment [17]. Other recommendations for healthcare worker recruitment include targeting medical students who have a rural background in educational programs, locating schools with health professional programs that are outside of major cities, exposing undergraduate students studying health-related topics to rural medicine, and designing continuing education programs that are adequate and accessible to providers living in rural areas [18].

The recruitment of healthcare providers in rural areas is a global issue [13,15]. However, little research has been conducted on healthcare provider recruitment in rural areas in the United States, specifically in a state like Idaho. Additionally, factors affecting the recruitment of healthcare providers in rural communities may vary across countries, regions, states, or cities. Therefore, there is a need to explore state- and community-specific data to support efforts to recruit providers to rural areas. The purpose of this exploratory study was to examine the strengths or challenges in the recruitment of rural healthcare providers in a rural and frontier community in northern Idaho.

## 2. Materials and Methods

### 2.1. Data Source and Participants

A cross-sectional questionnaire survey was used on a sample of healthcare providers practicing in a rural and frontier community in northern Idaho, which assessed factors related to healthcare provider recruitment. The Qualtrics survey platform was used for data collection. Links to the Qualtrics survey were distributed by a supervising physician and an administrator at the two local hospitals in the community who then disseminated the survey links to healthcare providers (i.e., anyone at the hospital providing direct patient care no matter their professional credential). The survey included a retooled version of the validated Nursing Community Apgar Questionnaire (NCAQ) [19] and a demographic questionnaire. Participants were offered a USD 25 Amazon gift card for completing the survey. This project was reviewed by the University Institutional Review Board and certified as exempt.

### 2.2. Instrumentation

#### 2.2.1. Modified Nursing Community Apgar Questionnaire (NCAQ)

The NCAQ [19] is a survey tool developed to identify strengths and challenges related to rural nurse recruitment and retention; minor modifications were made to ensure all healthcare providers (not just nurses) from the community could complete the survey [19]. The NCAQ is a 50-factor questionnaire, broken down into five classes of factors (i.e., geographic factors, economic factors, scope of practice factors, medical support factors, and facility and community support factors). Each class contains 10 factors. Each factor was measured using two Likert scales, which evaluated how advantageous or challenging a factor was in recruiting healthcare providers (major challenge = −2, minor challenge = −1, minor advantage = 1, major advantage = 2) and the importance of a factor in the recruitment of healthcare providers (very unimportant = 1, unimportant = 2, important = 3, very important = 4). For the advantage/challenge scale, positive mean scores indicate on average that respondents perceived those factors as advantages, while negative mean scores indicate that respondents on average perceived those factors as challenges. Mean scores on the importance scale that were rated as ≥ 3 mean that, on average, respondents perceived those factors as important, while mean scores < 3 mean that, on average, respondents perceived those factors as unimportant. To determine the highest-rated factors in terms of importance, advantage, and Agar score (importance × advantage), classes and factors were compared.

#### 2.2.2. Demographic Questionnaire

The demographic questionnaire included sex, age, ethnicity, and geographical location where most of childhood was spent (>50%). Clinical practice demographic factors included profession, clinical credentials, years of clinical practice experience, school attended for medical training, and location of residency training.

### 2.3. Data Analysis

Data collected from August to December 2021 were inputted into Qualtrics survey software (Qualtrics, Provo, UT, USA; https://www.qualtrics.com). Statistical analysis was performed using SAS version 9.4 (SAS Institute, Cary, NC, USA). Categorical factors were presented with frequencies and percentages, while continuous factors were presented with means and standard deviations. Recruitment factors were measured using Likert scales which were presented using means and standard deviations. These factors were evaluated and analyzed according to the methods described by the creators of the NCAQ [19]. Factors were measured according to their perceived importance, as well as the extent to which they were considered an advantage or challenge. Scores regarding the advantage/challenge of each factor were multiplied by scores for importance to calculate the ‘Apgar’ score (Advantage/Challenge × Importance) [19]. Apgar scores have a possible range of −8 to 8.

## 3. Results

A total of 50 healthcare providers completed the recruitment survey. Respondents were, on average, 42.7 years of age (SD = 13.5, range: 24–74) and had 14.0 years of clinical practice experience (SD = 12.4, range: 0–44). The highest proportions of respondents were female (52.0%), white (90.0%), lived in an area classified as a town (32.0%), and were physicians (50.0%). A full breakdown of provider demographics is presented in Table 1.

### 3.1. Modified Nursing Community Apgar Questionnaire

#### 3.1.1. Advantages and Challenges

Table 2 presents the overall mean advantage/challenge scores for our sample. The highest class was identified as facility and community support, with mean scores ranging from −1.26 to 2.00 (major advantage = 2, minor advantage = 1, minor challenge = −1, major challenge = −2). Across classes, the factors identified as the top 10 advantages were perceived fiscal stability (M = 0.25), community need/support (M = 0.27), minor trauma (M = 0.30), climate (M = 0.38), continuing medical education (CME) benefit (M = 0.42), teaching (M = 0.43), emergency/stabilization care (M = 0.43), leadership (M = 0.60), recreational opportunities (M = 1.29), and medical reference resources (M = 2.00); all these were determined to be the most important factors. The factor identified as the biggest advantage was medical reference resources (M = 2.00). The top advantages were from the facility and community support (community need/support, medical reference resources, leadership), scope of practice (emergency/stabilization care, minor trauma, teaching), geographic (climate, recreational opportunities), and economic (CME benefit, perceived fiscal stability) classes.

The top ten challenges were specialist availability (M = −1.30), access to the larger community (M = −1.29), electronic medical records (M = −1.26), nursing workforce (M = −1.22), language support services (M = −1.17), televideo support (M = −1.10), shopping and other services (M = −1.08), allied mental health workforce (M = −1.01), housing (M = −1.00), and mental health (M = −0.95). The factor that was the biggest challenge was specialist availability (mean score = −1.3), and 8 out of these 10 challenges had a score below −1, indicating that they were beyond the “minor challenge” level (minor challenge = −1). More of these top challenges were from the medical support class (nursing workforce, specialist availability language support services, allied mental health workforce) than any other class. However, there were also factors from the geographic (access to a larger community, housing, and shopping other services), facility and community support (electronic medical records, televideo support), and scope of practice (mental health) classes.

#### 3.1.2. Importance

Table 2 presents the overall mean scores for the importance scale. Medical support was rated as the most important class, where 9 out of 10 factors were rated above 3.00 (score of 3 = important). Across all classes (e.g., geographic, economic), the top ten factors for the importance scale were electronic medical records (M = 3.39), allied mental health workforce (M = 3.39), nursing workforce (M = 3.48), call/practice coverage (M = 3.48), leadership (M = 3.48), stability of workforce (M = 3.52), emergency/stabilization care (M = 3.61), spousal satisfaction (M = 3.63), salary (M = 3.63), and perception of quality (M = 3.70). The lowest ten factors for the importance scale were language support services (M = 2.48), mid-level supervision (M = 2.48), demographics (M = 2.54), social networking (M = 2.67), moonlighting opportunities (M = 2.68), office GYN opportunities (M = 2.70), competition (M = 2.75), administration (M = 2.78), access to the larger community (M = 2.83), and part-time opportunities (M = 2.87).

#### 3.1.3. Overall Apgar Scores

Table 2 presents the overall mean Apgar scores for our sample. The facility and community support class was rated as the most impactful mean Apgar scores, which ranged from −4.27 to 6.26. The top 10 factors for the community Apgar scores (most important advantages), across all classes, were perceived fiscal stability (M = 0.79), community need/support (M = 0.88), minor trauma (M = 0.93), climate (M = 1.10), CME benefit (M = 1.31), teaching (M = 1.32), emergency/stabilization care (M = 1.55), leadership (M = 2.09), recreational opportunities (M = 4.19), and medical reference resources (M = 6.26). The 10 most important challenges (lowest mean Apgar scores) were electronic medical records (M = −4.27), nursing workforce (M = −4.25), specialist availability (M = −4.19), access to the larger community (M = −3.65), allied mental health workforce (M = −3.42), spousal satisfaction (M = −3.34), housing (M = −3.29), televideo support (M = −3.20), shopping and other services (M = −3.15), and language support services (M = −2.90).

## 4. Discussion

As a predominantly rural state, Idaho has a severe shortage of healthcare providers and limited medical resources for its residents [20]. A number of factors have been identified as being related to healthcare providers’ decision to practice in rural areas [10,11,21]. However, little research has been conducted on the recruitment of healthcare providers in Idaho, a predominately rural state. The purpose of the current study was to explore factors of healthcare provider recruitment in a rural and frontier community in northern Idaho to identify strengths and weaknesses. The findings of this study provide a valuable contribution to the body of knowledge on provider recruitment in rural areas. Furthermore, little research on this topic has presented community-level findings in a U.S. state, and these are essential to addressing variations among communities.

In our sample, medical support was identified as the most important class because it included more factors rated as important (e.g., nursing workforce) than any other class. However, factors from all other classes (economic, facility and community support, geographic, and scope of practice) contained factors rated as important. Additionally, the facility and community support factor was rated as the highest advantage and had the highest Apgar scores, indicating the most important advantages.

Prior research identified medical support factors as important to healthcare recruitment in rural areas [12]. An Australian study using the NCAQ reported the nursing workforce as one of the most important advantages for the recruitment of providers [12]. In contrast, our study observed the nursing workforce as one of the most important challenges to recruitment. Furthermore, another Australian study reported the scope of practice factor emergency care as a challenge, although it was not identified as a top factor [13]. However, emergency/stabilization care in our study was identified as a top advantage. Such differences in findings may be due to structural and medical practice differences between healthcare in Australia compared to the United States [22].

Facility and community support factors have been reported as important to provider recruitment in prior research [14]. In a study conducted on physicians’ perceptions of telemedicine, rural physicians more frequently used telemedicine, with higher use being associated with higher reported value to physician support in the community [14]. Moreover, in a study conducted in rural Canada, rural physicians indicated a greater utilization of telemedicine compared to urban physicians [14]. The increased use of telemedicine was also linked to a higher perceived value of the use of telemedicine in supporting community healthcare [14]. Our current study identified televideo support as one of the most important challenges to recruitment, which may be influenced by the greater use of telemedicine by rural healthcare providers [14].

Prior research supports economic factors as important in the recruitment of healthcare providers [15,16]. In a study conducted in Manitoba, Canada, physicians reported that income was very important regarding whether or not they would select a position in a rural area [15]. Furthermore, another study assessing the effectiveness of financial incentive programs reported that 93% of physicians who received financial incentives said that finances were a moderate or major factor in their choice to apply to the program [16]. Similarly, in the current study, economic class factors “CME benefits” and “perceived fiscal stability” were among the most important advantages to recruitment in our study.

In the current study, spousal satisfaction and access to the larger community were identified as important challenges to recruitment among our sample. Geographic factors such as these have also been reported in previous studies as important factors to the recruitment of rural providers [13,23]. In a study conducted in rural Australia, which utilized the NCAQ to evaluate the recruitment of nurses, participating nurses reported low levels of satisfaction for spousal support and access to the larger community [13]. These results are consistent with the finding in our sample of these factors being challenges in their community. Additionally, a study conducted in the U.S. using the NCAQ reported spousal satisfaction as one of the top 3 reasons reported regarding the recruitment of physicians in rural areas [23], which is consistent with our finding that this factor was reported as important by the participants in our sample.

### Limitations and Future Research

This study is not without limitations. One of the limitations was the small sample size. This was due to the limitations of funds and the small population of healthcare providers to survey in the rural and frontier community in northern Idaho. Additionally, the self-reported nature of our survey may have resulted in survey-related biases, such as social desirability bias [24]. It is also unknown what factors would have been reported as important advantages or challenges by providers who elected to not participate in the survey and how this would have affected the results. Providers who did not respond to the survey may have reported different factors as important advantages or challenges. We were also limited based on the categorization of our questions regarding the profession of the participating provider. Nearly one-fifth (18.0%) reported a profession not listed in the question (marked “other”), which restricts our knowledge regarding to whom these findings can be generalized. This study was also conducted on a single community and, therefore, the results are limited in their generalizability to other communities, the state of Idaho, or the United States. Future research should be conducted on other rural communities in northern Idaho and across Idaho to develop more generalizable results for factors that may act as strengths or challenges to the recruitment of rural providers.

## 5. Conclusions

The current study identified multiple factors as potentially important challenges and advantages for provider recruitment in a rural and frontier community in northern Idaho. Overall, respondents reported multiple geographic, facility and community support, economic, and medical support factors as important advantages and challenges to the recruitment of healthcare providers in rural areas. In particular, factors related to facility and community support, as well as medical support, are of particular importance and may act as strengths or challenges to healthcare professional recruitment in rural settings. Additionally, individual factors identified as important challenges should be considered for further research, including electronic medical records, nursing workforce, specialist availability, access to the larger community, allied mental health workforce, spousal satisfaction housing, televideo support, shopping and other services, and language support services.

Policymakers and healthcare administrators seeking to improve the recruitment of providers in rural areas may elect to target such factors for policy development and hiring strategies to improve provider recruitment. Healthcare administrators may utilize the findings of this study, along with findings from related research, to select medical support and facility/community support factors that may be modified to increase the recruitment of providers. For factors not easily modifiable at an administrator level, healthcare administrators may elect to advocate for policy change through collaboration with policymakers who also have an interest in improved recruitment of healthcare providers in rural areas.

Due to the limited research available regarding U.S. studies, and the limited scope of this research on recruitment of providers in rural areas, future research among other rural populations is necessary to make locational comparisons between findings. Additionally, future studies should implement a qualitative research methodology to understand these findings in greater depth.

## Figures and Tables

**Table 1:** Estimated population characteristics of survey respondents.

Characteristic	M (SD)
Age	42.7 (13.5)
Years of clinical practice	14.0 (12.4)
	**N (%)**
Sex	
Male	23 (46.0)
Female	26 (52.0)
Prefer not to answer	1 (2.0)
Ethnicity	
American Indian or Alaska Native	2 (4.0)
Hispanic or Latino or Spanish Origin	1 (2.0)
Native Hawaiian or Other Pacific Islander	1 (2.0)
White	45 (90.0)
Prefer not to answer	3 (6.0)
Childhood Geographical Location	
Large city (500,000 residents or more)	5 (10.0)
Suburb of a large city	3 (6.0)
City of a moderate size (50,000 to 500,000 residents)	7 (14.0)
Suburb of a moderate size city	3 (6.0)
Small city (10,000 to 50,000 residents–other than a suburb)	5 (10.0)
Town (2500 to 10,000 residents–other than a suburb)	16 (32.0)
Small town (population less than 2500 residents)	11 (22.0)
Profession	
Physician	25 (50.0)
Physician Assistant	3 (6.0)
Nurse Practitioner	2 (4.0)
Nurse	8 (16.0)
Pharmacist	1 (2.0)
Public Health Professional/Healthcare Administrator	1 (2.0)
Other	9 (18.0)
Unknown	1 (2.0)

**Table 2:** Mean scores for advantage/challenge, importance, and the community Apgar score.

Class	Factor	Advantage/ Challenge ^1^	Importance ^2^	Apgar Score ^3^
Economic	Part-time Opportunities	−0.38	2.87	−1.09
Economic	Loan Repayment	0.21	3.25	0.68
Economic	Salary (Amount)	−0.29	3.63	−1.05
Economic	Signing Bonus/Moving Expenses	−0.09	3.25	−0.29
Economic	Length of Contract Flexibility	0.17	3.13	0.53
Economic	Perceived Fiscal Stability	0.25	3.17	0.79
Economic	Production Incentive	0.25	3.00	0.75
Economic	Retirement Package	0.08	3.25	0.26
Economic	CME * Benefit	0.42	3.13	1.31
Economic	Competition	0.17	2.75	0.47
Facility & community support	Physical plant and equipment	−0.48	3.35	−1.61
Facility & community support	Plans for capital investment	−0.60	3.09	−1.85
Facility & community support	Electronic medical records	−1.26	3.39	−4.27
Facility & community support	Leadership	0.60	3.48	2.09
Facility & community support	Televideo support	−1.10	2.91	−3.20
Facility & community support	Community need/support	0.27	3.26	0.88
Facility & community support	Welcome and recruitment program	0.09	2.91	0.26
Facility & community support	Medical reference resources	2.00	3.13	6.26
Facility & community support	Delegated patient services	0.05	3.18	0.16
Facility & community support	Moonlighting opportunities	0.23	2.68	0.62
Geographic	Access to larger community	−1.29	2.83	−3.65
Geographic	Demographics: underserved/pay or mix	−0.75	2.54	−1.91
Geographic	Housing (availability/affordability)	−1.00	3.29	−3.29
Geographic	Schools	−0.83	3.25	−2.70
Geographic	Social networking	−0.83	2.67	−2.22
Geographic	Recreational opportunities	1.29	3.25	4.19
Geographic	Spousal satisfaction (education, work, general)	−0.92	3.63	−3.34
Geographic	Shopping and other services	−1.08	2.92	−3.15
Geographic	Climate	0.38	2.92	1.10
Geographic	Perception of Community	−0.08	3.04	−0.24
Medical support	Perception of quality	−0.17	3.70	−0.63
Medical support	Stability of physician workforce	−0.57	3.52	−2.01
Medical support	Specialist availability	−1.30	3.22	−4.19
Medical support	Nursing workforce	−1.22	3.48	−4.25
Medical support	Mid-level provider workforce	0.09	3.09	0.28
Medical support	Ancillary staff workforce	−0.22	3.26	−0.72
Medical support	Pharmacy services	−0.30	3.04	−0.91
Medical support	Allied mental health workforce	−1.01	3.39	−3.42
Medical support	Language support services	−1.17	2.48	−2.90
Medical support	Call/practice coverage	−0.57	3.48	−1.98
Scope of practice	Obstetrics: prenatal care	−0.21	3.09	−0.65
Scope of practice	Obstetrics: deliveries/C-section	−0.21	3.13	−0.66
Scope of practice	Inpatient care	0.09	3.35	0.30
Scope of practice	Emergency/stabilization care	0.43	3.61	1.55
Scope of practice	Minor trauma (casting/suturing)	0.30	3.09	0.93
Scope of practice	Office GYN procedures	0.13	2.70	0.35
Scope of practice	Mental health	−0.95	3.04	−2.89
Scope of practice	Mid-level supervision	0.17	2.48	0.42
Scope of practice	Teaching	0.43	3.09	1.32
Scope of practice	Administration	0.09	2.78	0.25

^1^ mean scores were derived from a −2 to 2 scale where −2 = major challenge, −1 = minor challenge, 1 = minor advantage, and 2 = major advantage. ^2^ mean scores were derived from a 1 to 4 scale where 1 = very unimportant, 2 = unimportant, 3 = important, and 4 = very important. ^3^ Apgar scores are the mathematical product of the importance scores and the advantage/challenge scores (Importance × Advantage/Challenge = Apgar). * CME = continuing medical education.

## Data Availability

The datasets presented in this study are available on request from the corresponding author.
